# Pushing the envelope: the feasibility of using a mailed contrast sensitivity test to prioritise cataract waiting lists

**DOI:** 10.1038/s41433-024-03081-6

**Published:** 2024-05-27

**Authors:** Eleonora Bianchi, Peter F. Reddingius, Mehal Rathore, Dan Lindfield, David P. Crabb, Pete R. Jones

**Affiliations:** 1 Glaucoma Services, Royal Surrey County Hospital NHS Foundation Trust, Guildford, UK; 2 Department of Optometry and Visual Sciences, School of Health and Psychological Sciences, City, University of London, London, EC1V 0HB England

**Keywords:** Prognostic markers, Prognosis, Risk factors

## Abstract

**Background:**

Cataract waiting lists are growing globally. Pragmatic, cost-effective methods are required to prioritise the most urgent cases. Here we investigate the feasibility of using a third-party pen-and-paper contrast sensitivity, CS, test (SpotChecks^TM^), delivered by mail, and performed by patients at home unsupervised, to flag eyes requiring surgery.

**Methods:**

Pen-and-paper CS tests were mailed to 233 people waiting for a cataract assessment, along with a prepaid return envelope (cross-sectional study). Response rates were tabulated (stratified by age, sex and socioeconomic status), and test scores analysed to see how well the home tests predicted which eyes were listed subsequently for surgery. A subset of patients (*N* = 39) also underwent in-person follow-up testing, to confirm the accuracy of the home data.

**Results:**

Forty-six percent of patients responded (216 eyes). No gross differences were observed between respondents and non-respondents, either in terms of age, sex, socioeconomic status, or geographic location (all *P* > 0.05). The home-test CS scores predicted which eyes were subsequently listed for surgery, with an AUROC {±CI_95%_} of 0.69 {0.61–0.76}. Predictive performance was further-improved when machine learning was used to combine CS scores with letter acuity, extracted from patients’ medical records (AUROC {±CI_95%_} = 0.77 {0.70–0.83}). Among 39 patients who underwent follow-up testing, home CS scores were correlated with various measures made in clinic: biometry signal-to-noise (*P* = 0.032), LogMAR acuity, Pelli-Robson CS and SpotChecks CS (all *P* < 0.001).

**Conclusions:**

Mailing patients pen-and-paper CS tests may be a feasible, 'low-tech' way of prioritising patients on cataract waiting lists.

## Introduction

Patients and physicians agree that cataracts should ideally be treated within 3 months of diagnosis and that waiting times longer than 6 months are excessive [1–4]. Waits longer than 6 months are also associated with reduced quality of life, and increased risk of depressive symptoms, falls, and other life-changing accidents [5–10]: often exacerbating the burden on healthcare services long-term [11].

Historically, many health services have struggled to meet these targets. In the last decade, for example, patients typically waited: 1.5 months (United States Medicare [12]) 1–6 months (mainland Europe [13]), 3 months (Scotland [14]), or 8 months (Australia [15]) for surgery—often with an additional 3–12 months [14, 16] wait for the initial pre-surgical assessment following referral.

And even the best-performing services will face unprecedented strain as societies age, with the demand for cataract surgery forecast to increase by 50% over the next 20 years [17]. Even with more efficient practices (e.g. via same-day assessments and surgery [18], simultaneous bilateral extractions [19–21], dedicated operating rooms [22], out-of-office hours slots for cataract surgery [23], or by foregoing surgery altogether in low-impact cases [24]), cataract waiting lists are only likely to grow globally.

In light of this, there have been calls to revisit the longstanding question of how best to manage cataract waiting lists [25, 26]. Clearly, more urgent cases should be prioritised for treatment [1]. And while precisely how urgency should be calculated is a complex and contentious topic [27], one key determinant must be the severity of vision loss that the patient is currently experiencing. The question then becomes how to quantify patients’ current level of vision loss, in a way that is scalable and cost-effective—and does not further burden already overstretched health services?

'Telemedicine' may provide an answer: enabling patients to assess their own vision at home. The logistical hurdles are considerable, however. Providing digital testing equipment to millions of patients would be prohibitively expensive, and asking patients themselves (two thirds of whom are over 60 years old [28] and many with limited vision) to access and learn to use custom software using their own devices is unlikely to prove feasible, and risks 'Digital Exclusion' for a subset of the most vulnerable individuals [29].

In the present study we, therefore, took a novel, 'low tech' approach: examining whether it is feasible to simply post out a pen-and-paper assay of contrast sensitivity [30] (CS) to patients currently waiting for a cataract assessment. CS was preferred over visual acuity since CS is thought to be a more sensitive marker of cataract severity [31–33] (and of consequent vision-related disability [26]), and because in practical terms, CS—unlike acuity—does not require stringent control of viewing distance, allowing the test to be easily self-administered (e.g. while sat at a table).

Patients were prospectively mailed two CS tests (one per eye) and asked to return the completed tests via an enclosed prepaid envelope. For realism, our participants were not preprepared in any way. They had not been previously asked to participate in a study, forewarned by their clinician to expect a letter, trained how to use the test, or offered any support when completing the test, other than the enclosed written instruction.

Completion rates were tabulated, broken down by age, sex and socioeconomic status. Accuracy was assessed by comparing the results of home testing to various measures made subsequently in clinic (biometry, CS). Usefulness was assessed by evaluating how well the home test data predicted which eyes were subsequently listed for surgery (either when the home data were considered in isolation, or when a machine learning algorithm was used to combine them with other readily available sources of patient information).

## Methods

### Participants

Test packs were prospectively mailed to 233 individuals, selected at random from people on a waiting list for a pre-surgical cataract assessment at Royal Surrey County Hospital: a secondary care centre in south-east England. No attempt was made to target particular types of patients. However, as shown in the results, patients generally resided in affluent neighborhoods, and, in a random subsample of 39 patients, 100% self-reported as Caucasian. This study was approved by the NHS Health Research Authority (IRAS ID: #300328) and was conducted in accordance with the Declaration of Helsinki.

### Procedure

**Fig. 1:**
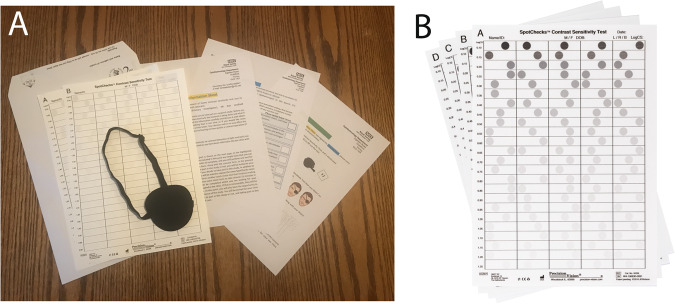
Methods. **A** Contents of the test pack posted to each patient (*see body text for details*). **B** Close-up of the SpotChecks test, including the circular targets that the user must circle or tick. There were six variants in total (A–F; only 4 of which are shown here). Patients were posted a random pair, with no duplication within patient. Note that the image has been enhanced for visibility—the spots in the bottom rows are not normally visible even to a normally sighted observer.

Each patient was mailed a test pack, the contents of which are shown in Fig. 1. This consisted of: (i) 2x SpotChecks tests (a pen-and-paper CS test, *detailed below*, to be completed monocularly, once per eye); (ii) an eye patch; (iii) a 3-page information sheet and consent form (mandated by research ethics); (iv) a prepaid return envelope; and (v) a set of instructions on how to perform the tests.

Patients were asked to complete one CS test per eye (fellow eye patched), and to return the completed tests using the prepaid envelope provided. Crucially, patients were given no forewarning (were not told to expect the pack, or notified that a research study was taking place), were not issued with any reminders or encouragements to return the tests, were not incentivised in any way, and had never before been shown the test.

For all 233 patients (*N* = 466 eyes), demographic information was extracted from their medical records, in order to assess any systematic differences between respondents and non-respondents. This included age, sex, ocular and general medical history, and home postcode. Home postcode was also used to estimate their Index of Multiple Deprivation [34] [IMD]—a score that divides the UK into 32,844 parcels of land, and then ranks each based on various considerations such as local income, education, crime and living environment (1 = most deprived; 32,844 = least deprived).

Furthermore, for the 108 individuals (*N* = 216 eyes) who returned a completed home test, additional clinical information was collated retrospectively from their medical records following their subsequent cataract consultation, in order to assess the accuracy and utility of the home test data. This consisted of which eyes were listed for immediate surgery, biometry signal-to-noise ratios for each eye, and Snellen acuity scores for each eye.

Finally, 39 of these 108 patients (*N* = 78 eyes) were randomly selected to undergo detailed follow-up assessments (administered whilst waiting to see the consultant at their next cataract clinic appointment). They were asked to perform the SpotChecks test again, once per eye, under supervision, and also to perform a Pelli Robson contrast test once per eye. These data were used to further validate the accuracy of the home test scores.

### The SpotChecks™ contrast sensitivity test

The SpotChecks test (Fig. 1B—formerly called 'CamBlobs' [30]) consists of a single sheet of A4 paper onto which small spots of decreasing contrast are printed (Precision Vision, Woodstock, IL, USA). The patient’s task is to mark, with a pen or pencil, the location of each spot (e.g. by ticking, crossing, or circling). Each test is therefore single use, and retails at ~£1 ($1.25 USD) per sheet. The standard SpotChecks test was used, though subsequent to the present study a 'Low Vision' variant was also released.

Following the manufacturer’s recommendations, test score was calculated based on the contrast of the last successfully seen target, with counting stopped after the second missed target (see [35] for alternative scoring methods, none of which appear to alter the overall pattern of results substantively). The outcome is a single number: a CS value (in decibels), with larger values indicating better CS.

SpotChecks was used as it was, to our knowledge, the only such pen-and-paper CS test commercially available. The study authors have no relationship with Precision Vision (manufacturers of the SpotChecks test, and also the Pelli Robson reference measure). The tests were purchased at a fair market rate, and Precision Vision was not involved in any aspect of the study.

Note also that while we refer to SpotChecks test as 'low tech' this is not technically correct, as advanced printing methods and stringent quality controls are required to precisely fix the contrast of each ink spot. This is a marked difference from other paper-based tests such as the Moorfield Home Acuity Test [36], which is designed to be printed using ordinary home or office printers.

### Analysis

Data were analysed using standard inferential statistics and described using 95% confidence intervals computed using bootstrapping (*N* = 20,000; bias-corrected and accelerated method).

## Results

### Response rates

Of the 233 patients posted a test pack, 108 (46.4%) responded. All 108 respondents successfully enclosed two completed SpotChecks tests (one per eye; *N* = 216 eyes total). As shown in Supplemental Fig. S1, there were no obvious demographic differences between respondents and non-respondents, either in terms of age, sex, socioeconomic status, or geographic distance (all *P* > 0.1; see Table 1 for statistics). One patient who didn’t respond was subsequently reported as deceased, but no systematic inquiries were made into the circumstances of the other 124 non-respondents.

**Table 1:** Associated statistics for Supplemental Fig. S2.

Characteristics of patients who did/did not respond			
	Mean {SD} or %		Group comparison stats
	Non-respondents	respondents	
Age (years)	74.8 {12.6}	75.1 {9.3}	${t}_{231}=-0.22,P=0.823$
Distance from hospital (km)	12.4 {8.3}	11.6 {7.7}	${t}_{231}=0.79,P=0.428$
Index of multiple deprivation (rank)	25211 {2097}	25451 {1906}	${t}_{229}=-0.91,P=0.364$
Sex	53.6% female	54.6% female	${{{{{{\rm{\chi }}}}}}}_{(1,N=233)}^{2}=0.03,P=0.875$

No statistically significant differences were apparent between mean values from the two groups, either in terms of age, location, IMD or sex.

### Predicting future listing for cataract surgery

**Fig. 2:**
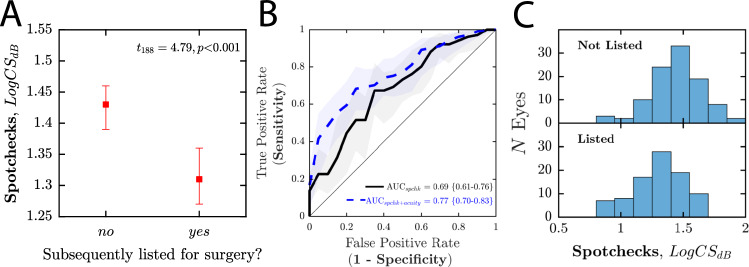
Ability of SpotChecks, performed at home, to predict which eyes were subsequently listed for surgery. **A** Mean [±95% confidence intervals] SpotChecks scores for eyes that were/were not subsequently listed for surgery, plus associated *t*-test values (see also Fig S2 for further analysis). **B** Receiver Operating Characteristics [ROCs] showing the ability of SpotChecks to predict which eyes were/were not subsequently listed for surgery. Shaded regions indicate the 95% confidence intervals. The black line indicates when SpotChecks data alone were used. The blue dashed line indicates when a Support Vector Machine was used to combine SpotChecks data and Snellen acuity scores (model trained and evaluated using leave-one-out analysis, using the following Matlab functions: fitcsvm.m, fitSVMPosterior.m, kfoldPredict.m, perfcurve.m). Numerical values show the Area Under the ROC [AUROC], plus 95% confidence intervals. **C** Histograms showing the distributions of raw SpotChecks scores.

Figure 2 shows how well the home CS scores predicted which eyes were subsequently listed for cataract surgery following the patient’s next consultation (NB: the consultant was blind to the results of the SpotChecks home-testing data when determining which eyes to list). Note that for these analyses only 95 of the 108 respondents were included (i.e. *N* = 190 eyes), as 13 patients had yet to attend an ophthalmic consultation at the time of writing. Two eyes were not listed for surgery, but were nevertheless scored as listed, as surgery was recommended but subsequently postponed (once by patient request; once due to more urgent medical complications).

Eyes listed for surgery tended (Fig. 2A), on average, to score significantly more poorly on SpotChecks (*t*_188_ = 4.79; *P* < 0.001), indicating that the result of the home test was associated with the need for cataract surgery (see Fig. 2C for raw scores).

More directly, Fig. 2B (black line) shows how well SpotChecks predicted which individual eyes were subsequently listed for surgery. The resultant classifier had a Sensitivity = 73% at a Specificity = 54%, with an overall Area Under the ROC {±CI_95%_} of 0.69 {0.61–0.76}. We also considered whether this score could be further improved by combining the SpotChecks data with other information easily obtainable from a patient’s medical record. For example, the dashed blue line in Fig. 2B illustrates the results of a machine learning classifier (Support Vector Machine) that combined SpotChecks scores with Snellen acuity. The model was trained and evaluated using a ‘leave one out’ technique and was found to improve performance by approximately 10%. Thus, the resultant classifier had a Sensitivity = 79% at a Specificity = 61%, with an overall Area Under the ROC {±CI_95%_} of 0.77 {0.70–0.83}. Adding other additional factors to the model (e.g. age, sex, biometry scores, a history of ocular disease [yes/no]) did not appear to further improve the classifier, though we did not explore this question exhaustively given the limited size of the data set (see Supplemental Fig. S3 for additional analyses).

### Agreement with other biomarkers

**Fig. 3:**
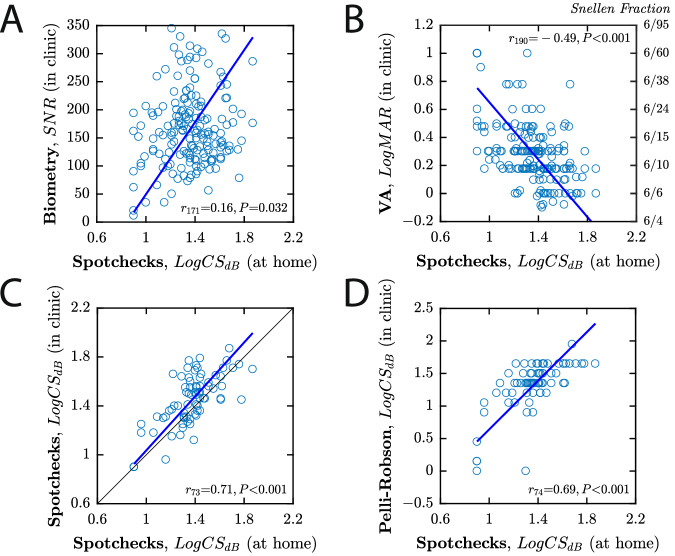
Scatterplots showing the agreement between SpotChecks performed at home, unsupervised and other biomarkers measured in clinic. **A** Biometry (Zeiss IOL Master 500) signal-to-noise ratio. **B** Letter distance acuity, measured using a Snellen chart at 3 m. **C** SpotChecks performed supervised, in clinic (see Supplemental Fig. S4 for associated Bland–Altman analysis). **D** Pelli Robson contrast sensitivity. In each case, each marker represents a single eye (Note that panels (**C**, **D**) have fewer data points as only a subset of 39 patients underwent a detailed follow-up assessment. Numbers also differ slightly between all four panels due to occasional missing data). Numerical values indicate Pearson correlation coefficients. Blue lines show the least-squares geometric mean regression slope.

As shown in Fig. 3, SpotChecks performed at home was weakly correlated with biometry signal-to-noise ratio [*r*_171_ = 0.16, *P* = 0.032; Fig. 3A], and negatively correlated with letter acuity [*r*_188_ = −0.49, *P* = 0.001; Fig. 3B]. A subset of patients (*N* = 39) also underwent a more detailed follow-up assessment. These data confirmed that SpotChecks performed at home were positively correlated with SpotChecks performed in clinic under supervision [*r*_73_ = 0.71, *P* < 0.001; Fig. 3C], and were also correlated with the results of the Pelli Robson letter chart: the clinical reference standard for CS [*r*_74_ = 0.69, *P* < 0.001; Fig. 3D].

### Patient feedback

Thirty-six of these patients were asked if they would have preferred the home test to be delivered digitally. 72% (26 of 36) said they preferred the pen-and-paper testing approach, while 14% would have preferred a digital test and 14% expressed no preference.

## Discussion

This pilot study demonstrates the feasibility of using a low-cost pen-and-paper CS test to prioritise those individuals most in need of cataract surgery.

### Response rate

The response rate was 46%. *Prima facie*, this figure may appear low. However, given how the tests were administered (with no patient selection, pre-warning, incentive, follow-up, or support), we actually consider it remarkably high. Prior research suggests that the rate of return could be further increased through relatively inexpensive measures such as automated reminders [37, 38] or financial incentives [39, 40] (e.g. lotteries). It should also be noted that a high return rate is not necessarily a prerequisite for this approach to be viable. Thus, given its low per-patient cost, and given that non-respondents are not necessarily disadvantaged (e.g. rather than being put to the back of the queue, non-respondents could be assigned randomly generated scores, leaving them no worse off than in the present prioritisation 'lottery'), it may be that the collection of additional data for patient prioritisation may be justified, even if the response-rate were low.

No gross differences were observed between respondents and non-respondents, either in terms of age, sex, socioeconomic status, or geographic location. However, this should be taken in the context of the relatively homogenous sample. Further research is required to identify whether specific demographics might be particularly well/ill-served by pen-and-paper home testing.

### Cost

The cost of pen-and-paper home testing was around £3 ($4 USD) per patient (incl. postage fees and test materials), not accounting for the staff time taken to prepare the outbound packs and score the returned tests. To minimise these staffing costs, we also developed a means of automatically scoring and transmitting test results using a smartphone camera – potentially obviating the need for patients to even post backtests. We intend to publish technical details of this software at a future date (*manuscript in preparation*).

### Other variables to consider when determining how to prioritise patients

Visual function is just one of the factors a clinician must consider when deciding how to prioritise patients. For example, when computing a prioritisation ’score' it may also be prudent to factor-in patient self-reports [2], general health, life expectancy, and the patient’s circumstances, including possible threats to independent living or employment. Exactly how to weigh these factors is outside the scope of the present work, and they are moral and political judgments as much as they are scientific questions.

Furthermore, even if just considering visual function, it is highly likely that other measures—in addition to the simple CS summary measure considered in the present study—would allow cataract severity to be more fully characterised. For example, in addition to CS, low contrast acuity, [31] disability glare, [41] visual search performance, [42] and stereopsis [43] have all been shown to be associated with degraded quality of life due to cataracts (indeed, often much more so than conventional measures of visual acuity [41, 43]). It is therefore extremely likely that by also collecting such measures (and/or structural information from photographs [44]), would allow more accurate decisions regarding patient prioritisation to be made. Whether the benefits would justify the additional costs is unknown at present, however.

### Study limitations and future work

The sample of the present study was relatively small (*N* = 233; versus 400,000 surgeries performed annually in England [45]). And while patients were randomly selected, the sample population was not widely representative—all being residents of a disproportionately affluent/Caucasian suburb of Greater London. That said, there is no specific reason to think that the results of the present study would not generalise to a larger and more diverse sample, particularly given that the test itself poses relatively few linguistic or cognitive demands (e.g. 4-year-old children have been shown capable of performing the SpotChecks test competently [35]).

### Limitations of the test

CS measurements can be affected by ambient lighting conditions [46]—and unlike with digital tests, there was no way of automatically recording what the illumination levels were, or of warning patients if their testing environment is inappropriate. [47] (In the present study patients were simply asked to perform the test in a 'well lit room'.) As the test was performed unsupervised, there was also no way of ensuring that patients patched the fellow eye correctly (or at all), or even of ensuring that the correct person took the test. And since the patient was not 'forced' to mark every response box on the page (see Fig. 1B), individuals may have chosen to stop responding altogether when they could no longer clearly discern the target: confounding confidence with visual ability [48]. All of these factors may have affected the accuracy and reliability of the SpotChecks data to some degree. However, the fact that the home tests showed good agreement with those performed subsequently in clinic (supervised) is encouraging: Suggesting that either patients can be relied upon to perform the tests at home appropriately, and/or that variations in home testing environments do not deleteriously affect the quality of the data substantively.

A potential limitation of the concept (of using home measures of visual function to prioritise patients) is that 'if such a system were to be implemented, there would be tremendous incentive for patients to artificially suppress their own visual function scores [49]. We did not see any evidence of such malingering in the present study, but if home testing were integrated into routine practice then careful efforts may indeed be required to detect and militate against anomalous results.

### On the benefits of pen and paper testing

We believe that pen-and-paper testing was particularly well-suited to the present use-case, since testing was required to be one off, self-administered, and performed at scale and since even a moderate level of test accuracy was expected to be sufficient. However, this should not be taken to imply that pen-and-paper tests should always be preferred. For example, digital apps may be better suited in situations where more detailed assessments of vision need to performed, where performance needs to be tracked over time (e.g. for disease monitoring [50, 51]), or where it is important to refer a patient for further testing (e.g. for mass screening [47]). Nonetheless, the present data suggest that when it comes to prioritising cataract waiting list, a simple pen-and-paper test appeared to have many attractive qualities: being easily scalable, low maintenance, acceptable to patients (though see [52]), and avoiding issues of Digital Exclusion [29].

## Conclusions

This study examined the feasibility of using a pen-and-paper CS test, administered by mail, and performed unsupervised at home, to help prioritise patients waiting for a pre-surgical cataract assessment. The data showed that around half (46%) of patients responded. And in those that did respond, the results of the home test were correlated with related measures made subsequently in clinic (biometry, acuity, CS). The home data were also reasonably predictive of which eyes were subsequently listed for surgery, particularly when combined with visual acuity scores extracted from patients’ medical records. Taken together, these results indicate that a low-tech, low-cost pen-and-paper test might feasibly be used to help inform the prioritisation of patients on cataract waiting lists, and complements a wider trend, both in ophthalmology and beyond, towards using 'asynchronous testing' to augment more conventional methods of patient assessment [53].

## Summary

### What was known before

- It is known that cataract waiting lists are long and growing and that tools are needed to intelligently prioritise patients.

### What this study adds

- This study demonstrates that mailing patients pen-and-paper vision tests may be a feasible, low tech way of prioritising patients on cataract waiting lists.
- This showcases a new, pragmatic means of managing cataract services.

## Supplementary information

Supplemental Material

## Data Availability

Anonymised data will be made available online. This includes all of the data reported in the present manuscript, with the exception of patient identifying information (date of birth, home address, medical history). This study demonstrates that cataract patients are willing and able to perform pen-and-paper vision testing at home, and the data provided can be used to identify eyes in need of surgery (thereby suggesting a pragmatic means of managing overstretched eye care services).
