# Supplementary material for: Pushing the envelope: the feasibility of using a mailed contrast sensitivity test to prioritise cataract waiting lists
Source: Eye (Lond). 2024 May 27;38(13):2568–74. doi: 10.1038/s41433-024-03081-6 (PMC11385213; doi:10.1038/s41433-024-03081-6)
Supplement: Supplementary file 1 — Supplemental Material [file 41433_2024_3081_MOESM1_ESM.pdf]

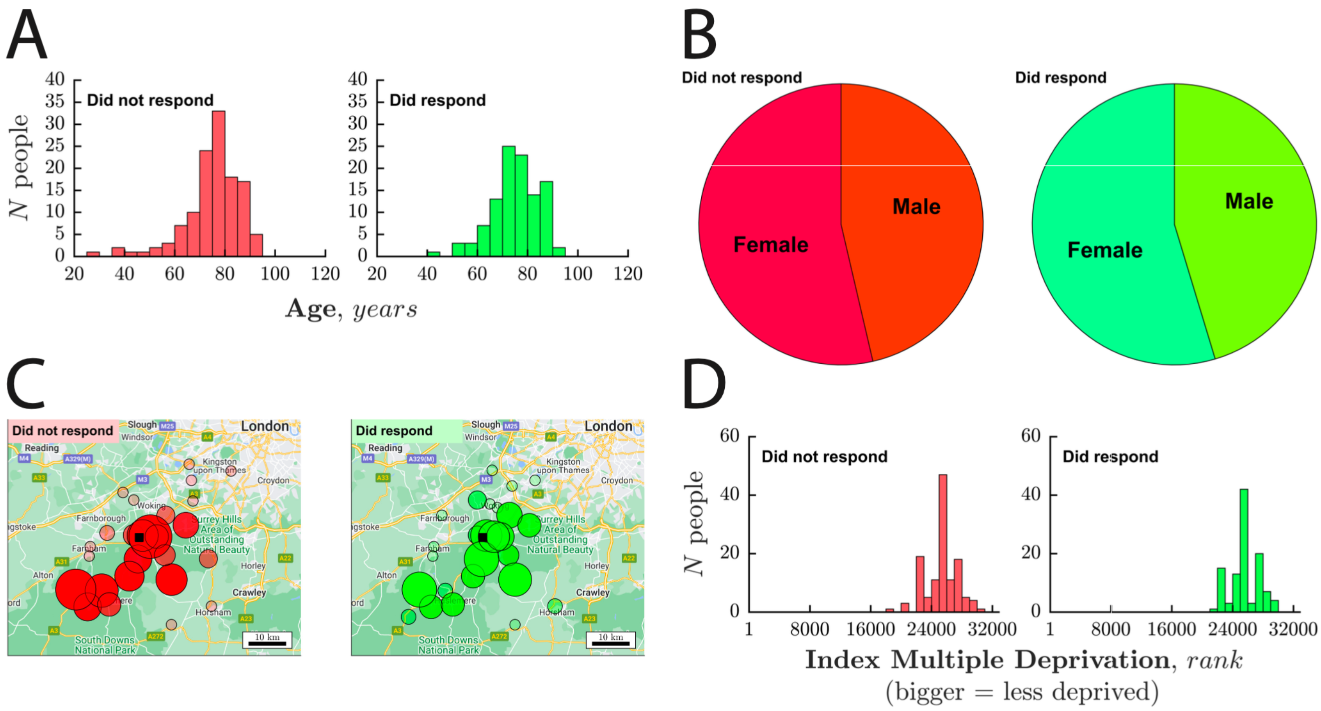


**Fig S1.** Demographic data for respondents (green) and non-respondents (red). See **Table 1** for associated statistics. In each panel, response rates are shown as a function of: ***(A)*** age; ***(B)*** sex; ***(C)*** geographic location (black square indicates the location of the hospital); ***(D)*** IMD (1 = most deprived; 32844 = least deprived).

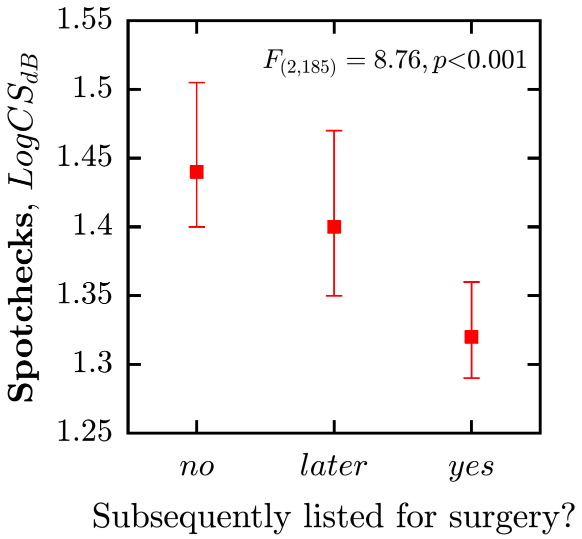


**Fig S2.** *Complementary analysis for **Fig 3A**of main manuscript*. Often eyes that were not immediately listed for surgery were subsequently listed (e.g., as the surgeon did not want to operate bilaterally). In the present figure eyes that fell under the “no” category are subdivided into “never” and “later”. Bars indicate mean [± 95% confidence intervals] SpotChecks scores. Numbers indicate associated one way ANOVA values.

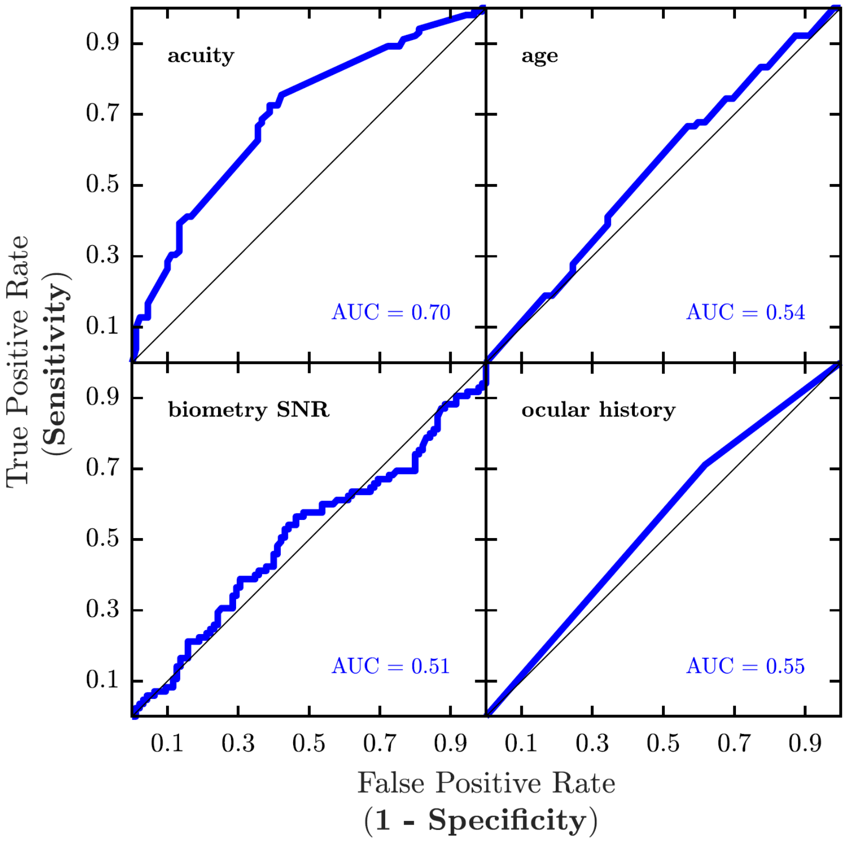


**Fig S3.** *Complementary analysis for Fig 3B of main manuscript*. This figure shows ROC curves, indicating the ability of age, sex, biometry signal-to-noise ratio, and a history of ocular disease [yes/no] to predict which eyes were subsequently listed for surgery (each considered independently in isolation).

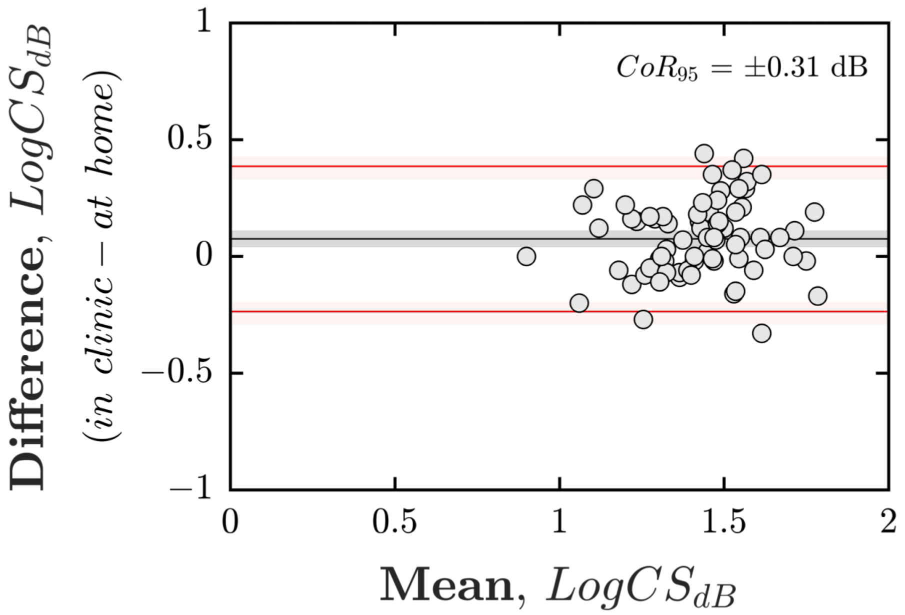


**Fig S4.** *Complementary analysis for Fig 4C of main manuscript*. This figure shows the results of a Bland-Altman analysis, comparing SpotChecks performed at home (unsupervised) and in the clinic (supervised), for the 39 patients who underwent enhanced follow-up testing. Each marker represents a single eye. The horizontal lines represent the mean agreement, plus lower and upper 95% limits of agreement. Shaded regions indicate 95% confidence intervals (computed using bootstrapping). The coefficient of repeatability was ± 0.31 $LogCS_{dB}$.
